# Mapping of SARS-CoV-2 spike protein evolution during first and second waves of COVID-19 infections in India

**DOI:** 10.2217/fvl-2021-0267

**Published:** 2022-06-03

**Authors:** Vijay Rani Rajpal, Shashi Sharma, Avinash Kumar, Samantha Vaishnavi, Apekshita Singh, Deepmala Sehgal, Mughdha Tiwari, Shailendra Goel, Soom Nath Raina

**Affiliations:** ^1^Department of Botany, Hansraj College, University of Delhi, Delhi, 110007, India; ^2^Virology Division, Defence Research & Development Establishment, Gwalior, Madhya Pradesh, 474002, India; ^3^Department of Botany, Vinoba Bhave University, Hazaribag, Jharkhand, 825319, India; ^4^Department of Botany, Central University of Jammu, Rahya Suchani (Bagla), Distt. Samba, Jammu and Kashmir, 181143, India; ^5^Amity Institute of Biotechnology, Amity University Uttar Pradesh, Sector 125, Noida, Uttar Pradesh, 201313, India; ^6^International Maize & Wheat Improvement Center (CIMMYT) Carretera México-Veracruz Km. 45, El Batán, Texcoco, 56237, México; ^7^ICMR-National Institute of Occupational Health (ICMR-NIOH), Meghaninagar, Ahmedabad, Gujarat, 380016, India; ^8^Department of Botany, University of Delhi, Delhi, 110007, India

**Keywords:** first and second COVID-19 wave of infections, immune evasion, mutation analysis, novel spike mutations, reduced neutralization, SARS-CoV-2

## Abstract

**Aim:** The aim of this study was to investigate the SARS-CoV-2 spike protein evolution during the first and second wave of COVID-19 infections in India. **Materials & Methods:** Detailed mutation analysis was done in 763 samples taken from GISAID for the ten most affected Indian states between March 2020 to August 2021. **Results:** The study revealed 242 mutations corresponding to 207 sites. Fifty one novel mutations emerged during the assessment period, including many with higher transmissibility and immune evasion functions. Highest number of mutations per spike protein also rose from 5 (first wave) to 13 (second wave). **Conclusion:** The study identified mutation-rich and no mutation regions in the spike protein. The conserved spike regions can be useful for designing future diagnostics, vaccines and therapeutics.

## Introduction

After the first reports of three cases of COVID-19 from Kerala, India on 30 January 2020, a significant number of positive COVID-19 cases from March 2020 onwards began registering. The first wave of COVID-19 infections peaked in September 2020 in India, with about 100,000 cases reported per day. From February 2021 onwards, India battled a brutal second wave of COVID-19 infections with severe consequences. The daily number of cases rose sharply and crossed the 400,000 mark and 4000 deaths every day in early May 2021 [1]. As compared to the first wave (highest 100,000 cases in September 2020), a sharp increase in positivity rate with a massive surge from 1.62% on 1 March 2021 to approximately 20% on 13 May 2021 was observed [1]. It created an emergency situation in the country with reduced supplies and increased deaths, especially in the young population [2,3].

Multiple factors might have potentially contributed to this sudden spike in cases, such as introduction of highly infectious SARS-CoV-2 lineages, complex interplay between mutant strains and violation of COVID-19 appropriate behavior. The roll out of vaccination programs from 16 January 2021 and the resultant natural selection pressure on the viral genomes might have induced novel mutations and contributed to the further evolution of circulating lineages. The emergence of novel SARS-CoV-2 lineage B.1.617.2 (Delta) in December 2020 also fueled the surge in daily infections and drove the second COVID-19 wave in India [4].

Since the emergence of COVID-19 disease in China in 2019, several variant lineages of SARS-CoV-2 including the current B.1.1.529 Omicron variant have emerged across the world. The variants have been classified as variants of concern (VOCs), variants of interest (VOIs), variants being monitored (VBMs) and variants of high concern (VOHCs) based on their differences in transmissibility, virulence or efficacy of diagnostics, vaccines and therapeutics [5]. Currently, the two listed variants of concern (VOCs) include Delta (B.1.617.2, AY lineages) and Omicron (B.1.1.529, BA lineages). Many other variant lineages including Alpha (B.1.1.7 and Q lineages), Beta (B.1.351 and descendent lineages), Gamma (P.1 and descendent lineages), Lambda (C.37 and descendent lineages), Mu (B.1.621 and B.1.621.1), Epsilon (B.1.427 and B.1.429), Eta (B.1.525). Iota (B.1.526) and Zeta (P.2) are being monitored (VBMs). There are no VOIs and VOHCs listed currently [5]. These novel SARS-CoV-2 lineages are better sustained than the Wuhan strain (clade O) due to fitness provided by the accumulated novel mutations that confer immune surveillance escape, high virulence, pathogenicity and better transmissibility resulting in deadlier resurgent outbreaks of infection globally. Many of these incurred mutations span the spike glycoprotein of SARS-CoV-2, wherein more than 5,000 mutations have already been reported [6,7].

**Figure 1.**
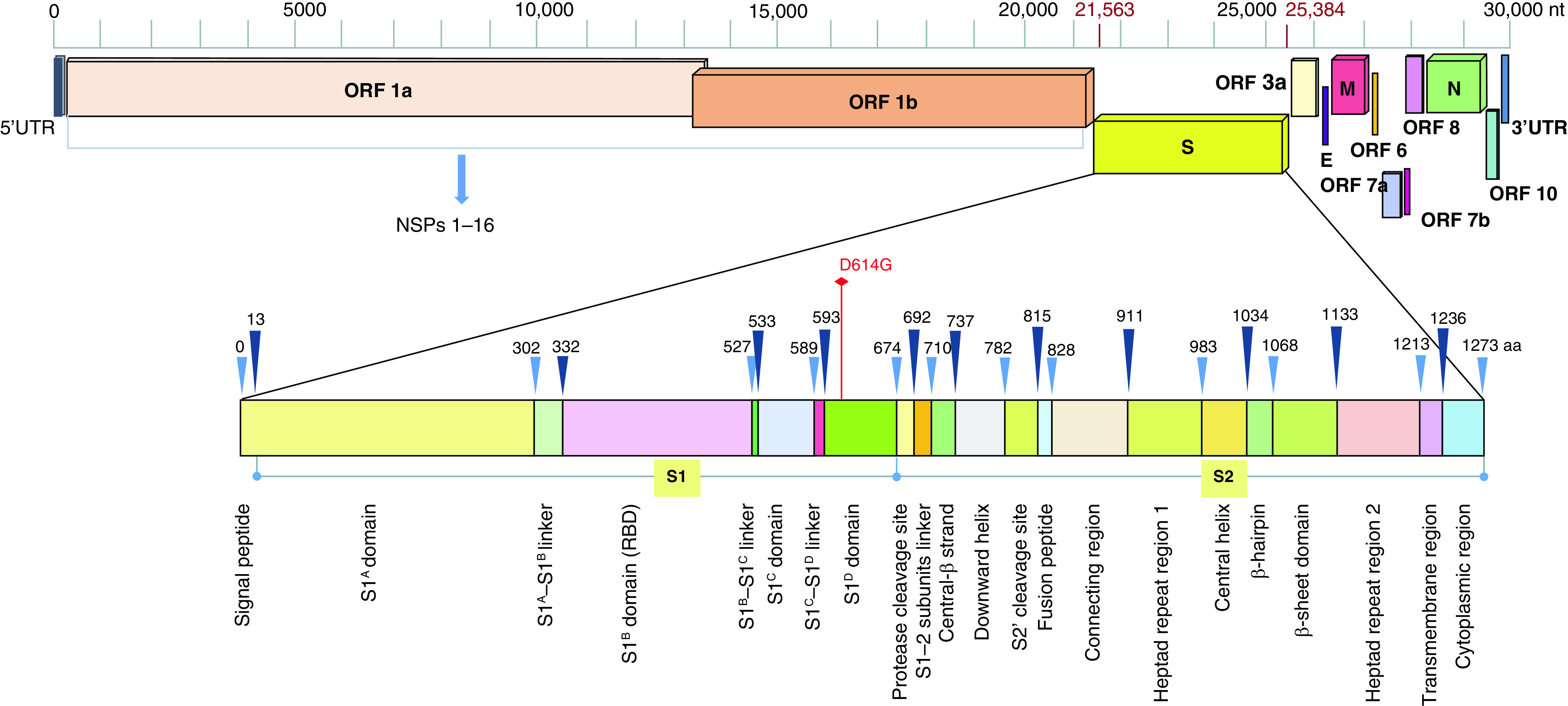
Detailed topology of SARS-CoV-2 with respect to nucleotide sequences and ORFs; domains and subdomains of spike protein.

The surface spike (S) protein present as small spikes on the SARS-CoV-2 surface is responsible for host cell receptor recognition and viral entry. It is encoded by the *S* gene (3821nt, position 21563–25384) and consists of 1273 amino acids [8]. At the N-terminus, S protein has a signal peptide (amino acids 1–13), which is followed by S1 and S2 subunits (Figure 1). The spike protein remains inactive in its native state. It is activated with help of proteases of the host cell membrane, specifically, a trans-membrane protease serine 2 (TMPRSS2) [10], that upon receptor recognition cleaves it into two subunits S1 and S2 at a furin-cleavage site [9,10]. S1 has the receptor binding domain (RBD) which is responsible for host cell angiotensin-converting enzyme 2 (ACE2) receptor recognition, interaction and binding. S2 domain brings about viral and host cell membrane fusion and enables viral entry into the host. S1 and S2 subunits are further divided into various domains and sub-domains (Figure 1).

In a rapidly evolving genome like SARS-CoV-2, the characterization of incurred mutations provides significant information for assessing the mechanisms linked to pathogenesis, immune evasion and viral drug resistance and also unravels useful insights into evolutionary patterns and spread of the virus. Furthermore, since the S protein has been an important target for designing anti-covid drugs, diagnostics and vaccines [11], the information about the conserved spike domains and sub-domains, if any, can be especially useful for designing the diagnostics, therapeutics and/or vaccines. In the present study we have undertaken a detailed analysis of Indian SARS-CoV-2 spike protein mutations in 763 randomly selected samples from ten Indian states deposited at Global Initiative on Sharing All Influenza Data (GISAID) [12] data base between March 2020 to August 2021, and compared it to prototype Wuhan sample (GenBank no. MN908947). The assessment period of about one and a half years includes both first and second waves of COVID-19 infections in India. The inferences of this study will be helpful in understanding the evolutionary trajectory of the virus and designing and supporting spike protein-based public health response.

## Materials & Methods

The nucleotide sequences of representative SARS-CoV-2 genomes were retrieved from GISAID database [12]. The 763 samples taken from March 2020 to August 2021 analyzed for mutation analysis in the present study included northern, southern, central and western Indian regions including Gujarat (G; n = 314), Maharashtra (M; n = 152), Karnataka (K; n = 67), Telangana (T; n = 43), Chhattisgarh (CH; n = 42), Uttar Pradesh (UP; n = 30), Madhya Pradesh (MP; n = 28), Haryana (H; n = 18), Rajasthan (R; n = 15) and the union territory of Delhi (D; n = 60). These ten states of India showed maximum positivity rate during the second wave of COVID-19 infections in India.

The mutations were analyzed on the basis of their presence or absence in different regions of spike protein. The mutation density was calculated as the number of mutations observed in a particular spike protein region divided by its size in base pairs, while frequency of a particular mutation was calculated as the number of samples showing a specific mutation divided by the total number of samples analyzed [13]. Three-dimensional crystal structure of spike protein available in the protein data bank (PDB; code: 6LZG) [14] was used to map the mutations located in the RBD.

## Results

Analysis of 763 spike protein mutations from March 2020 to August 2021 in ten Indian states revealed that as compared with lineage B.1 found between March to September 2020, B.1.1.7 lineage was detected from October 2020 to January 2021. During second wave of COVID-19 infections in February 2021 onwards, the number of circulating lineages rose to eight including the SARS-CoV-2 lineages B.1.1.7, B.1.351, B.1, B.1.525, B.1.36.17, B.1.617.1, B.1.617.2 and B.1.617.3. The massive spurt in cases and devastation observed during the second wave could be attributed to the presence of these multiple lineages especially VOC Delta and its sub-lineages. The states of Maharashtra, Kerala, Gujarat, Delhi, Karnataka, Chhattisgarh, Telangana among others included in this analysis reported maximum number of COVID-19 cases in India during the second wave of infections.

### Mutation analysis of the prevalent SARS-CoV-2 lineages in ten Indian states

#### Distribution of mutations in the spike protein

**Figure 2.**
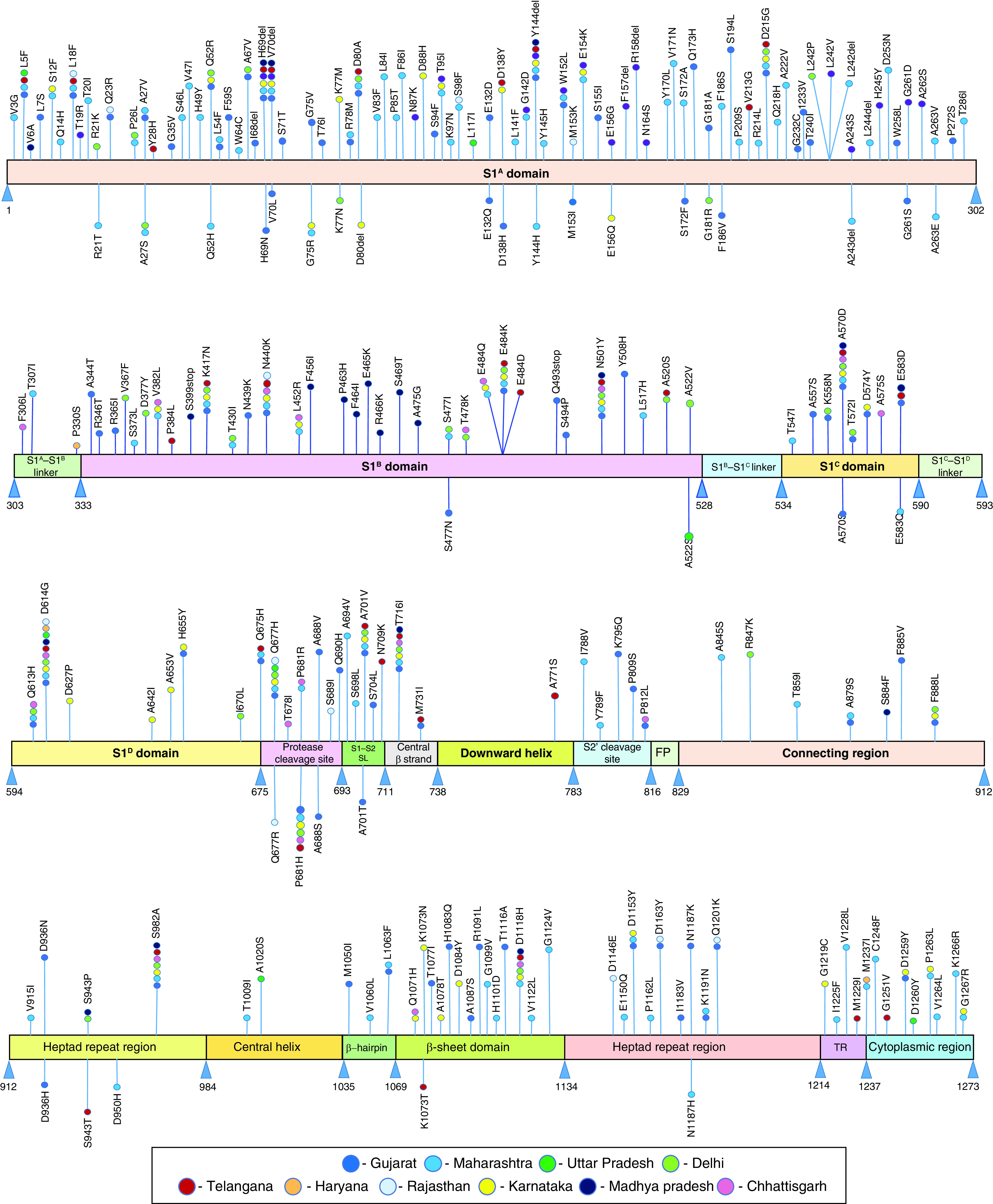
Distribution of mutations in human SARS-CoV-2 spike protein regions.

A total of 242 mutations corresponding to 207 sites in the spike protein were recorded during the assessment period (Table 1 & Figure 2). D614G was the only mutation present in all analyzed spike proteins between March 2020 to August 2021 from all the ten states. While, other prevalent mutations like N501Y and T716I (present in seven states), H69del, V70del, Y144del, N440K, P681H (in six states), D215G, K417N, E484K, Q671H and A701V (in five states), L5F, L18F, D80A, Q613H, Q675H (in four states) and Q52R, T95I, W152L, E154K, V382L, L452R, E484Q, A520S, F888L and D1135Y (in three states) showed differential distribution (Table 2 & Figure 2). There were 36 mutations that were present in 2 states each and the remaining mutations were represented individually in various states (Table 2 & Figure 2).

**Table 1.** Frequency of observed mutations in various domains of SARS-CoV-2 spike protein.

S no.	Mutation	Frequency	S no.	Mutation	Frequency	S no.	Mutation	Frequency
**S1^A^ domain (1-302)**			45	N87K	1	90	L242V	1
1	V3G	1	46	D88H	1	91	L242P	1
2	L5F	7	47	S94F	2	92	L242del	1
3	V6A	1	48	T95I	16	93	A243S	1
4	L7S	3	49	K97N	1	94	A243del	1
5	S12F	6	50	S98F	3	95	L244del	1
6	Q14H	2	51	L117I	1	96	H245Y	1
7	L18F	19	52	E132D	1	97	D253N	1
8	T19R	1	53	E132Q	1	98	W258L	1
9	T20I	1	54	D138Y	2	99	G261D	1
10	R21K	1	55	D138H	1	100	G261S	1
11	R21T	2	56	L141F	1	101	A262S	1
12	Q23R	5	57	G142D	6	102	A263V	1
13	P26L	2	58	Y144del	27	103	A263E	1
14	A27V	2	59	Y144H	1	104	P272S	1
15	A27S	3	60	Y145H	1	105	T286I	1
16	Y28H	1	61	W152L	5	**S1^A^-S1^B^ linker (303-332)**		
17	G35V	1	62	M153I	3	106	F306L	1
18	S46L	1	63	M153K	1	107	T307I	1
19	V47I	1	64	E154K	27	108	P330S	1
20	H49Y	1	65	S155I	1	**S1^B^ domain (333-527)**		
21	Q52R	5	66	E156G	1	109	A344T	1
22	Q52H	1	67	E156Q	1	110	R346T	2
23	L54F	41	68	F157del	1	111	R365I	1
24	F59S	1	69	R158del	1	112	V367F	1
25	W64C	1	70	N164S	1	113	S373L	1
26	A67V	4	71	Y170L	1	114	D377Y	1
27	I68del	1	72	V171N	1	115	V382L	4
28	H69del	23	73	S172A	1	116	P384L	1
29	H69N	1	74	S172F	1	117	S399stop	1
30	V70del	23	75	Q173H	1	118	K417N	14
31	V70L	1	76	G181A	2	119	T430I	2
32	S71T	1	77	G181R	1	120	N439K	1
33	G75V	1	78	F186S	1	121	N440K	15
34	G75R	4	79	F186V	1	122	L452R	38
35	T76I	1	80	S194L	1	123	F456I	1
36	K77M	4	81	P209S	1	124	P463H	1
37	K77N	1	82	V213G	1	125	F464I	1
38	R78M	7	83	R214L	3	126	E465K	1
39	D80A	14	84	D215G	14	127	R466K	1
40	D80del	3	85	Q218H	2	128	S469T	1
41	V83F	1	86	A222V	1	129	A475G	1
42	L84I	1	87	G232C	1	130	S477I	2
43	P85T	1	88	I233V	1	131	S477N	6
44	F86I	1	89	T240I	2	132	T478K	3
133	E484Q	31	**S1-S2 subunit linker (693-710)**			**β-sheet domain (1069-1133)**		
134	E484K	24	171	A694V	1	205	Q1071H	8
135	E484D	1	172	F698L	1	206	K1073N	1
136	Q493stop	2	173	A701V	16	207	K1073T	1
137	S494P	4	174	A701T	16	208	T1077I	1
138	N501Y	75	175	S704L	3	209	A1078T	1
139	Y508H	2	176	N709K	1	210	H1083Q	17
140	L517H	1	**Central-β strand (711-737)**			211	D1084Y	4
141	A520S	4	177	T716I	57	212	A1087S	1
142	A522S	1	178	M731I	2	213	R1091L	1
143	A522V	1	**Downward helix (738-782)**			214	G1099V	1
**S1^B^-S1^C^ linker (528-533)**			179	A771S	2	215	H1101D	9
**S1^C^ domain (534-589)**			**S2’ cleavage site (783-815)**			216	T1116A	1
144	T547I	1	180	I788V	1	217	D1118H	59
145	A557S	1	181	Y789F	1	218	V1122L	1
146	K558N	4	182	K795Q	1	219	G1124V	1
147	A570D	60	183	P809S	1	**Heptad repeat region 2 (1134-1213)**		
148	A570S	1	184	P812L	33	220	D1146E	1
149	T572I	5	**Fusion peptide (816-828)**			221	E1150Q	1
150	D574Y	2	**Connecting region (829-911)**			222	D1153Y	5
151	A575S	1	185	A845S	1	223	P1162L	1
152	E583D	14	186	R847K	1	224	D1163Y	5
153	E583Q	1	187	T859I	1	225	I1183V	1
**S1^C^-S1^D^ linker (590-593)**			188	A879S	3	226	N1187K	1
**S1^D^ domain (594-674)**			189	S884F	1	227	N1187H	2
154	Q613H	6	190	F885V	1	228	K1191N	5
155	D614G	697	191	F888L	6	229	Q1201K	3
156	D627P	1	**Heptad repeat region 1 (912-983)**			**Transmembrane region (1214-1236)**		
157	A642I	1	192	V915I	1	230	G1219C	1
158	A653V	1	193	D936N	1	231	I1225F	1
159	H655Y	3	194	D936H	1	232	V1228L	1
160	I670L	1 or all	195	S943P	2	233	M1229I	1
**Protease cleavage site (675-692)**			196	S943T	3	**Cytoplasmic region (1237-1273)**		
161	Q675H	11	197	D950N	2	234	M1237I	2
162	Q677H	41	198	D950H	1	235	C1248F	1
163	Q677R	2	199	S982A	58	236	G1251V	1
164	T678I	2	**Central helix (984-1034)**			237	D1259Y	2
165	P681R	33	200	T1009I	1	238	D1260Y	1
166	P681H	70	201	A1020S	1	239	P1263L	2
167	A688V	1	**β hairpin (1035-1068)**			240	V1264L	1
168	A688S	3	202	M1050I	2	241	K1266R	1
169	S6891	1	203	V1060L	1	242	G1267R	2
170	Q690H	5	204	L1063F	3			

Frequency of observed mutations in various domains shown in bold along with their amino acid positions

**Table 2.** Distribution of mutation types and sites in SARS-CoV-2 spike proteins in Indian states.

S no.	State	S proteins (n)	Mutations (n)	Mutation types and sites
1	Gujarat	314	96	L5F (3), L7S (3), L18F (4), Q23R (4), A27V (1), G35V (1), Q52R (1), **L54F (41)**, F59S (1), A67V (1), I68del (1), **H69del (14)**, H69N (1), **V70del (13)**, V70L (1), S71T (1), G75V (1), T76I (1), **R78M (6)**, D80A (4), S94F (2), E132D (1), E132Q (1), D138H (1), **Y144DEL (15)**, W152L (3), S155I (1), S172F (1), Q173H (1), G181A (1), F186V (1), S194L (2), D215G (4), G232C (1), I233V (1), T240I (2), W258L (1), G261S (1), P272S (1), A344T (1), R346T (2), R365I (1), K417N (4), N439K (1), N440K (4), **S477N (6)**, **E484K (5)**, Q493stop (2), S494P (1), **N501Y (20)**, Y508H (2), D574Y (1), A557S (1), K558N (3), **A570D (15)**, A570S (1), T572I (4), **E583D (14)**, Q613H (2), **D614G (302)**, H655Y (2), Q675H (2), **Q677H (21)**, **P681H (22)**, A688S (3), A688V (1), Q690H (6), **A701T (16)**, **A701V (5)**, S704L (3), **T716I (15)**, M731I (1), K795Q (1), P809S (1), **P812L (31)**, A879S (2), F885V (1), F888L (1), D936H (1), D936N (1), **S982A (15)**, M1050I (2), L1063F (2), T1077I (2), **H1083Q (17)**, A1087S (1), R1091L (1), T1116A (1), D1153Y (1), D1163Y (2), I1183V (1), N1187K (1), K1191N (4), Q1201K (1), D1259Y (1), M153I (3)
2	Maharashtra	152	106	V3G (1), L5F (3), **S12F (5)**, Q14H (1), **L18F (12)**, T20I (1), R21T (2), P26L (1), A27V (1), A27S (2), S46L (1) V47I (1), H49Y (1), Q52H (1), L54F (2), W64C (1), H69del (2), V70del (2), G75R (1), R78M (1), D80A (4), V83F (1), L84I (1), P85T (1), F86I (1), **T95I (12)**, K97N (1), S98F (1), L141F (1), G142D (1), Y144H (1), **Y144del (5)**, Y145H (1), W152L (1), **E154K (20)**, Y170L (1), V171N (1), S172A (1), F186S (1), P209S (1), R214L (3), D215G (4), Q218H (2), A222V (1), L242del (1), A243del (1), L244del (1), D253N (1), A263V (1), A263E (1), T286I (1), T307I (1) S373L (1), V382L(1), **K417N (5)**, T430I (1), N440K (1), L452R (2), S477I (1), **E484K(7)**, **E484Q (21)**, **N501Y (14)**, L517H (1), T547I (1), **A570D (9)**, E583Q (1), Q613H (2), **D614G (148)**, **Q675H (5)**, **Q677H (12)**, **P681H (10)**, **P681R (17)**, A694V (1), S698L (1), A 701V (4), **T716I (9)**, I788V (1), Y789F (1), A845S (1), T859I (1), A879S (1), V915I (1), D950H (1), **S982A (9)**, T1009I (1), V1060L (1), L1063F (1), G1099V (1), **H1101D (8)**, **D1118H (9)**, V1122L (1), G1124V (1), E1150Q (1), D1153Y (1), P1162L (1), N1187H (2), K1191N (1), I1225F (1), V1228L (1), M1237I (2), C1248F (1), P1263L (1), V1264L (1), K1266R (1), G1267R(1)
3	Karnataka	67	44	S12F (1), Q52R (3), **H69del (5)**, **V70del (5)**, G75R (1), K77M (4), D80del (1), D88H (1), T95I (1), D138Y (1), **Y144del (5)**, E154K (4), E156Q (1), D215G (3), V382L (2), K417N (3), **N440K (5)**, **L452R (5)**, **E484K (6)**, **E484Q (5)**, **N501Y (12)**, **A570D (7)**, D574Y (1), **D614G (52)**, D627P (1) A642I (1), A653V (1), H655Y (1), Q677H (3), **P681H (11)**, A701V (3), **T716I (9)**, F888L (3), **S982A (7)**, **Q1071H (5)**, K1073N (1), A1078T (1), D1084Y (4), **D1118H (7)**, D1153Y (2), G1219C (1), D1259Y (1), P1263L (1), G1267R (1)
4	Delhi	60	35	R21K (1), P26L (1), A27S (1), Q52R (1), A67V(3), K77N (1), D80A (1), G181R (1), D215G (1), L242P (1), V367F (2), D377Y (1), K417N (1), T430I (1), S477I (1), T478K (1), E484K (4), **N501Y (11)**, A520S (1), A522V (1), K558N (1), **A570D (12)**, T572I (1), Q613H (1), **D614G (51)**, I670L (1), Q677H (1), **P681H (8)**, A701V (1), **T716I (7)**, R847K (1), F888L(2), S943P(1), **S982A (10)**, **D1118H (10)**
5	Chhattisgarh	43	38	T19R (1), H69del (1), V70del (1), N87K (1), T95I (3), **G142D (5)**, Y144del (1), W152L (1), E154K (3), E156G (1), F157del (1), R158del (1), N164S (1), L242V (1), A243S (1), H245Y (1), G261D (1), A262S (1), F306L (1), V382L (1), N440K (2), **L452R (7)**, T478K(2), **E484Q (5)**, **N501Y (14)**, **A570D (14)**, A575S (1), Q613H (1), **D614G (41)**, T678I (2), **P681H (15)**, **P681R (7)**, **T716I (14)**, P812L(2), D950N (2), **S982A (15)**, Q1071H (3), **D1118H (14)**
6	Telangana	42	33	L5F (1), L18F (1), Y28H (1), H69del (2), V70del (2), D80A (2), Y144del (2), D138Y (1), V213G (1), D215G (2), P384L (1), K417N (1), N440K (2), E484D (1), E484K (2), **N501Y (5)**, A520S (3), A570D (3), E583D (1), **D614G (35)**, Q675H (1), P681H (4), A701V (2), N709K (1), T716I (3), M731I (1), A771S (2), S982A (3), S943T (1), K1073T (1), D1118H (3), M1229I (1), G1251V (1)
7	Madhya Pradesh	31	21	V6A (1), H69del (1), V70del (1), Y144del (1), S399stop (1), F456I (1), P463H (1), F464I (1), E465K (1), R466K (2), S469T (1), A475G (1), N501Y (1), A570D (1), E583D (1), **D614G (26)**, T716I (1), S884F (1), S943P (1), S982A (1), D1118H (1)
8	Uttar Pradesh	20	7	L5F (1), L117I (1), A522S (1), **D614G (15)**, Q677H (1), A1020S (1), D1260Y (1)
9	Haryana	18	3	**D614G (15)**, P330S (1), M1237I (1)
10	Rajasthan	16	12	L18F (2), Q23R (1), S98F (2), M153K (1), N440K (1), **D614G (12)**, Q677H (2), Q677R (3), S689I (1), D1146E (1), D1163Y (3), Q1201K (2)

Occurrences are indicated in parenthesis; 5 to 9 occurrences represented in bold; 10 and above are represented as bold and underlined.

#### Distribution of mutations among domains & subdomains of spike protein

The observed 242 mutations were found scattered through all the domains and subdomains of the spike protein except the S1^B^–S1^C^ and S1^C^–S1^D^ linkers, and the fusion peptide domains wherein no mutations were found (Table 1 & Figure 2). Mutations that were observed in just a single sample were termed as unique. The highest number of mutations (105) with 43.4% occurred in S1^A^ domain with 81 mutations listed as unique. It was followed by 35 mutations found in S1^B^ domain (14.5%) with 21 unique mutations and 15 mutations in β-sheet domain (6.9%) with 13 unique mutations (Table 1 & Figure 2).

#### Frequent spike protein mutations

As many as 170 out of 242 observed mutations were found to be unique. 28 mutations that appeared in more than 10 samples each were listed as frequent mutations. These frequent mutations with number of their occurrences and corresponding mutation frequency given in a decreasing order in parenthesis included: D614G (697; 0.92), N501Y (75; 0.099), P681H (70; 0.092), A570D (60; 0.079), D1118H (59; 0.078 ), S982A (58; 0.077), T716I (57; 0.075), Q677H (41, 0.054), L54F (41, 0.054), L452R (38; 0.05), P812L (33; 0.043), P681R (33; 0.043), E484Q (31; 0.041), E154K (27; 0.036), Y144del (27; 0.036), E484K (24; 0.031), H69del (23; 0.03), H70del (23; 0.03), L18F (19; 0.025), H1083Q (17; 0.022), A701V (16; 0.021), A701T (16; 0.021), S94F (16; 0.021), N440K (15; 0.02), D80A (14; 0.018), D215G (14; 0.018), K417N (14; 0.018) and Q675H (11; 0.014; Table 1). The remaining mutations showed anywhere between two to ten occurrences (Tables 1 & 2). The mutations showing five to nine occurrences are represented as bold, while 10 and above occurrences as bold and underlined in Table 2.

#### Mutation density in different regions of spike protein

**Figure 3.**
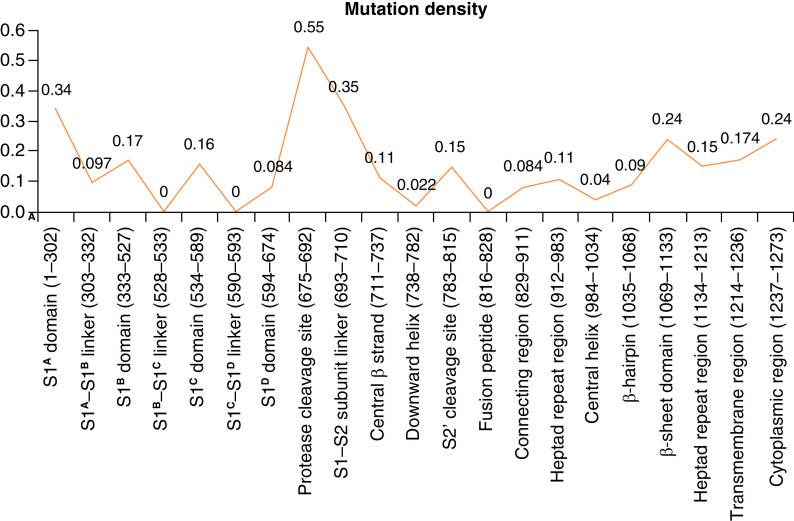
Mutation density in human SARS-CoV-2 spike regions in the analyzed samples.

The region-wise mutation density in the human SARS-CoV-2 spike protein is depicted in Figure 3. The highest mutation density (0.55) was observed in protease cleavage site, followed by 0.35 in S1–S2 subunit linker region, 0.34 in S1^A^ domain, 0.24 each in cytoplasmic region and β-sheet domain. The S1^B^ domain that forms the receptor binding domain and the transmembrane region showed a moderate mutation density of 0.17 each in the analyzed samples.

#### Spike protein sites with multiple mutation types

**Figure 4.**
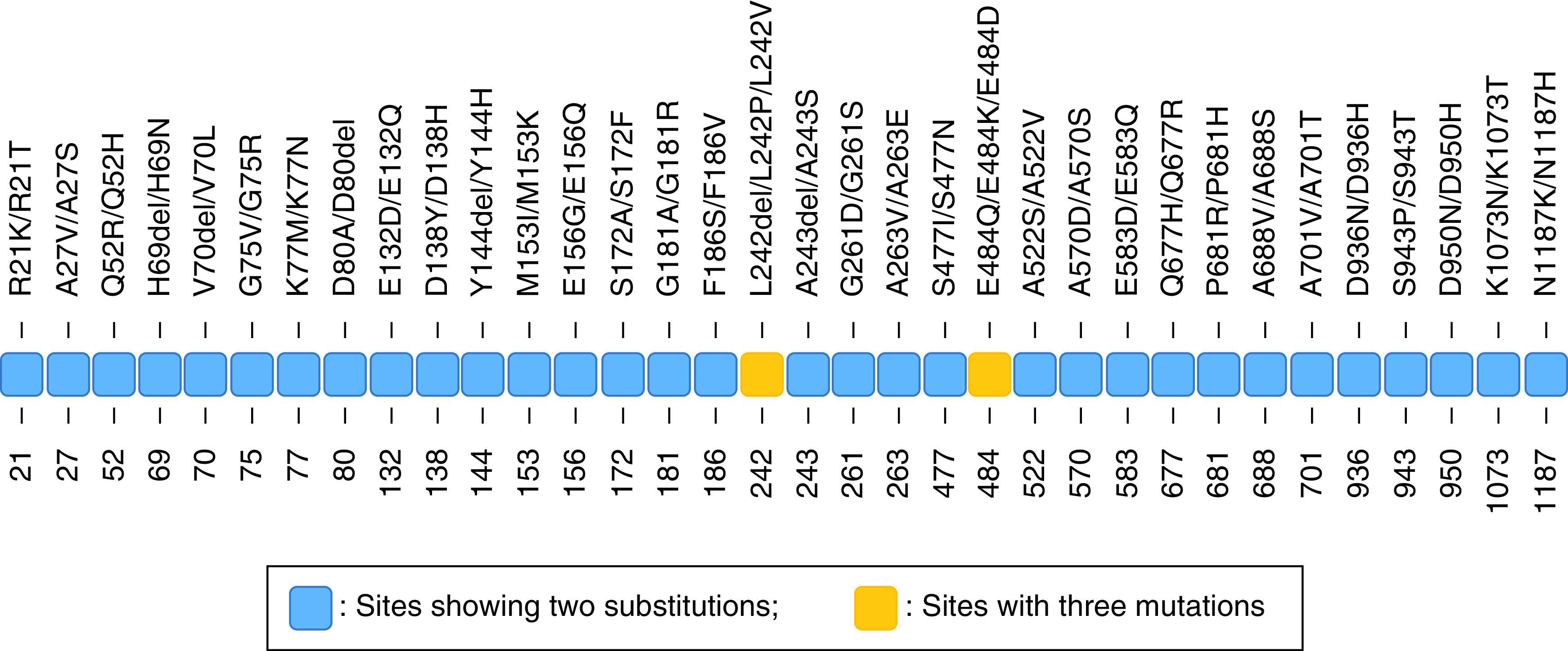
Human SARS-CoV-2 spike protein sites with multiple amino acid substitutions in ten Indian states.

We observed 34 sites in the analyzed spike proteins that harbored two or three different mutations at the same position in the spike protein sequence. Two sites 242 and 484 displayed three mutations each. At the former site, the amino acid leucine (L) was either deleted or changed to amino acid valine (V) or proline (P), while in the latter, glutamic acid (E) changed to either glutamine (Q), lysine (K) or aspartic acid (D). At the remaining 32 sites, two changes each were observed (Figure 4).

#### Spike proteins with maximum mutations

Seven hundred thirteen Indian human SARS-CoV-2 spike proteins carried from one to thirteen mutations. The highest number of mutations (13) were observed in spike protein from state of Maharashtra (B.1.351; EPI_ISL_1704062) followed by 11 each in Gujarat (B.1.1.7; EPI_ISL_1544115) and Chhattisgarh (B.1.617.2; EPI_ISL_1731755), 10 each in Telangana (B.1.1.7; EPI_ISL_1672391) and Karnataka (B.1.1.7; EPI_ISL_747244), 9 each in Delhi (B.1.351; EPI_ISL_1716786) and Madhya Pradesh (B.1; EPI_ISL_1251265), respectively. The parenthesis indicate the lineage description and representative GISAID accession number of the analyzed sample.

#### Mapping of observed mutations in RBD of SARS-CoV-2 spike protein

**Figure 5.**
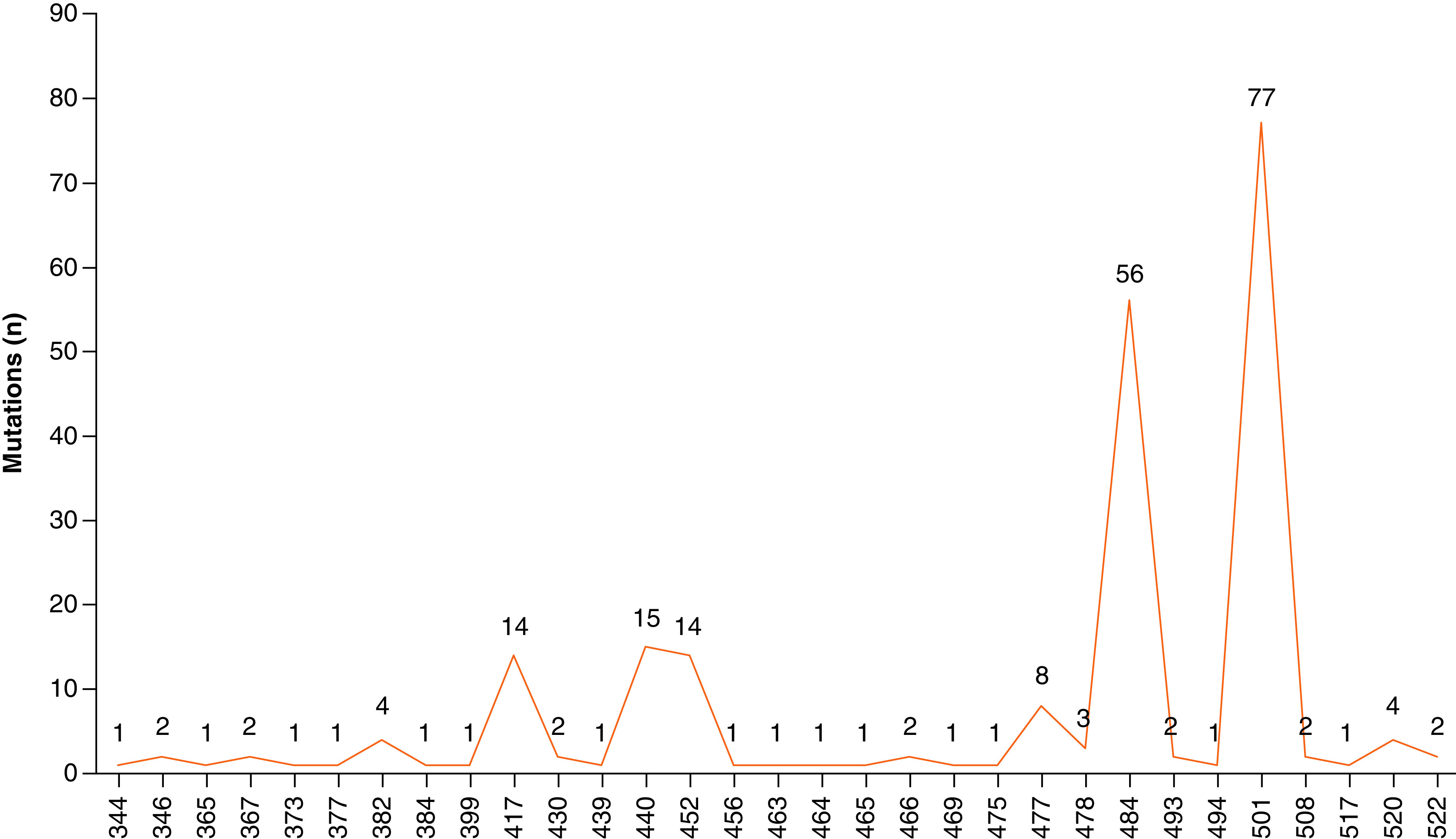
Mutations at 31 sites in the receptor binding domain of SARS-CoV-2 spike protein.

**Figure 6.**
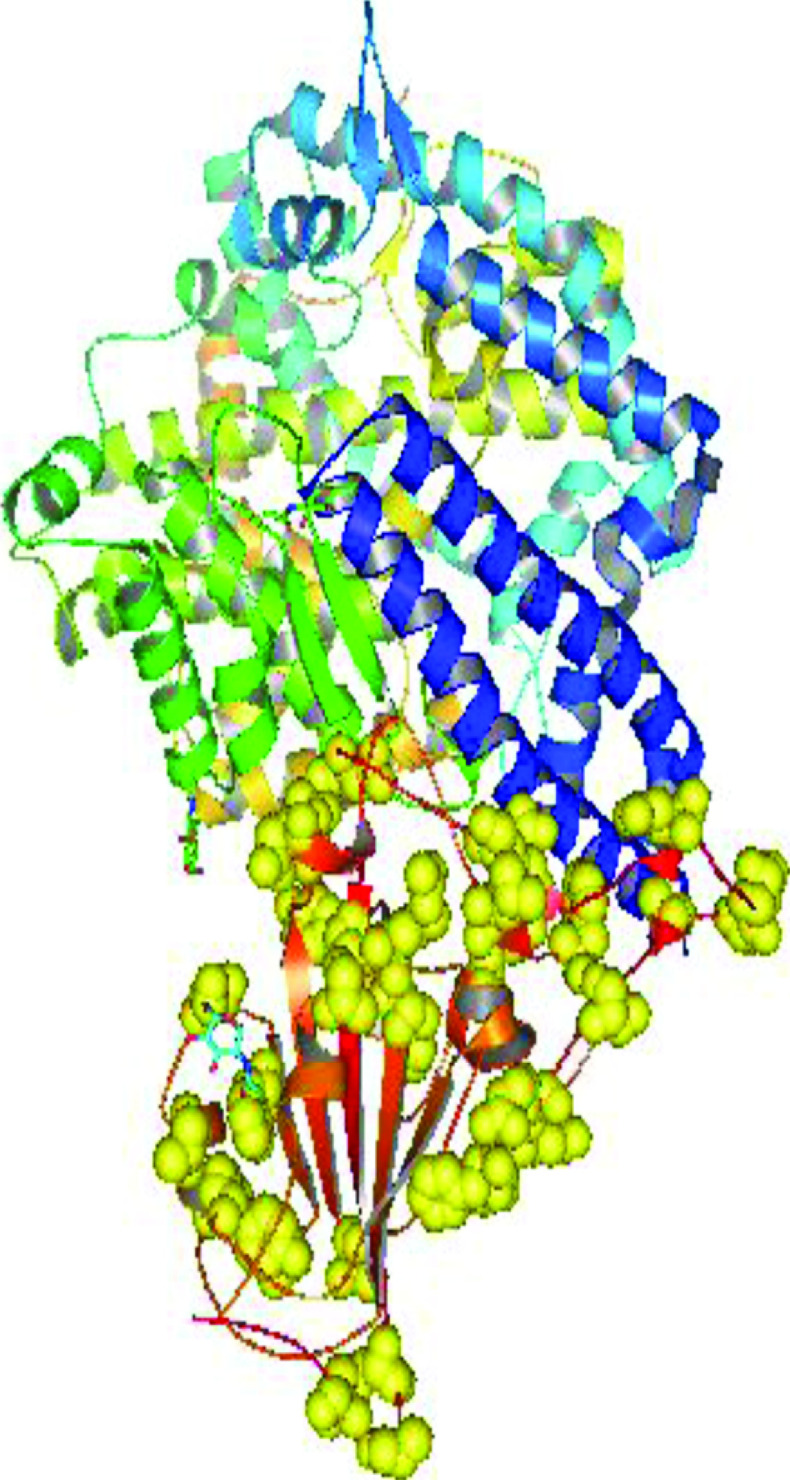
Mapping of 35 mutations on the crystal structure of spike protein receptor binding domain complexed with ACE2 receptor. PDB code: 6LZG; shown in yellow balls.

The RBD of spike protein interacts with host ACE2 receptor to mediate viral entry. The RBD region of spike proteins from ten different states revealed 35 distinct mutation types located at 31 distinct RBD sites as A344T, R346T, R365I, V367F, S373L, D377Y, V382L, P384L, S399stop, K417N, T430I, N439K, N440K, L452R, F456I, P463H, F464I, E465K, R466K, S469T, A475G, S477I, S477N, T478K, E484D, E484K, E484Q, Q493stop, S494P, N501Y, Y508H, L517H, A520S, A522S, and A522V (Table 2 & Figure 5). Mapping of these 31 mutations was done with a three dimensional crystal structure of human SARS-CoV-2 RBD complexed with ACE2 receptor (PDB code : 6LZG) as shown in Figure 6. Six of ten analyzed states except Karnataka, Chhattisgarh, Haryana and Rajasthan showed unique mutations that showed up as A344T, R346T, R365I, N439K, Q493stop, S494P and Y508H in Gujarat; S373L and L517H in Maharashtra; V367F, D377Y and A522V in Delhi; P384L and E484D in Telangana; S399stop, F456I, P463H, F464I, E465K, R466K, S469T, and A475G in Madhya Pradesh and A522S in Uttar Pradesh, respectively (Table 2).

The state-wise mutation types and sites of human SARS-CoV-2 spike protein in the RBD according to geographical locations is included in Table 2. The five most frequent mutations sites with occurrences in various states included 501 (G, M, K, D, CH, T and MP), 484 (G, M, K, D, CH, and T), 440 (G, M, K, D, CH and T), 452 (M, K, and CH) and 417 (G, M, K, D and T), respectively. A maximum number of mutants were observed at position 501 in the RBD of spike protein. This site harbored N501Y mutation that was present in 77 spike proteins from seven states (G, M, K, D, CH, T and MP). Site 484 with 56 mutations included mutants wherein amino acid E changed to K in 24 samples, to Q in the rest (Figure 5). We observed 50 spike proteins representing clade O (Wuhan isolate) with no mutations observed in them relative to the human SARS-CoV-2 spike protein reference sequence from Wuhan, China. These samples were present in all the analyzed states except Chhattisgarh. The maximum nine samples each were present in Gujarat and Delhi, while Haryana had the minimum number as three. Interestingly, amidst the presence of so many mutant lineages, clade O was visible in India throughout the assessment period from March 2020 until August 2021.

#### Samples without D614G

Although mutation D614G was ubiquitously registered in samples from all the ten states, there were 16 samples from five states *viz.* Gujarat (3; EPI_ISL_483869, EPI_ISL_483870 and EPI_ISL_586542), Karnataka (8; EPI_ISL_486398, EPI_ISL_486409, EPI_ISL_486408, EPI_ISL_486389, EPI_ISL_515961, EPI_ISL_515967, EPI_ISL_1595890 and EPI_ISL_1595875), Chhattisgarh (2; EPI_ISL_1173164 and 1 EPI_ISL_173162), Telangana (2; EPI_ISL_431101 and EPI_ISL_ 850514) and Madhya Pradesh (1; EPI_ISL_476840) which did not possess the most widely occurring mutation D614G. These samples were visible between March 2020 to July 2020 (Table 1). All the samples in remaining five states, and samples from aforementioned states after July 2020 contained D614G mutation either singly or in combination with other mutations.

#### Preferential site specific positive selection pattern for spike amino acid substitutions

Interestingly, we observed a preferential site specific positive selection pattern for particular amino acid substitutions in the spike protein. For instance, there were 11 sites (95, 240, 286, 307, 430, 547, 572, 678, 716, 859 and 1009), where amino acid threonine (T) always preferentially changed to isoleucine (I; 23 occurrences) across all the analyzed samples. T95I and T716I of these were the most frequent mutation types and sites. Only at three sites *viz.* 19 (Chhattisgarh), 478 (Chhattisgarh) and 1116 (Gujarat), T was observed to change to arginine (R), K and alanine (A), respectively (Table 2). Likewise, amino acid Q changed to histidine (H) at seven sites (14, 52, 218, 613, 675, 677 and 1071) in 13 occurrences. Q677H was the most frequent mutation type and site followed by Q613H. Except for one instance of Q677R mutation (karnataka), Q always changed to H at the 677 position across the states. Another preferential substitution was amino acid methionine (M) to I, observed at four sites (731, 1050, 1229 and 1237) with M731I being the most frequent mutation type and site in three occurrences. Phenyl alanine (F) to I (456 and 464) and Q to R (52 and 677) substitutions were observed at the mentioned two sites each (Table 2).

### State-wise mutation analysis

**Figure 7.**
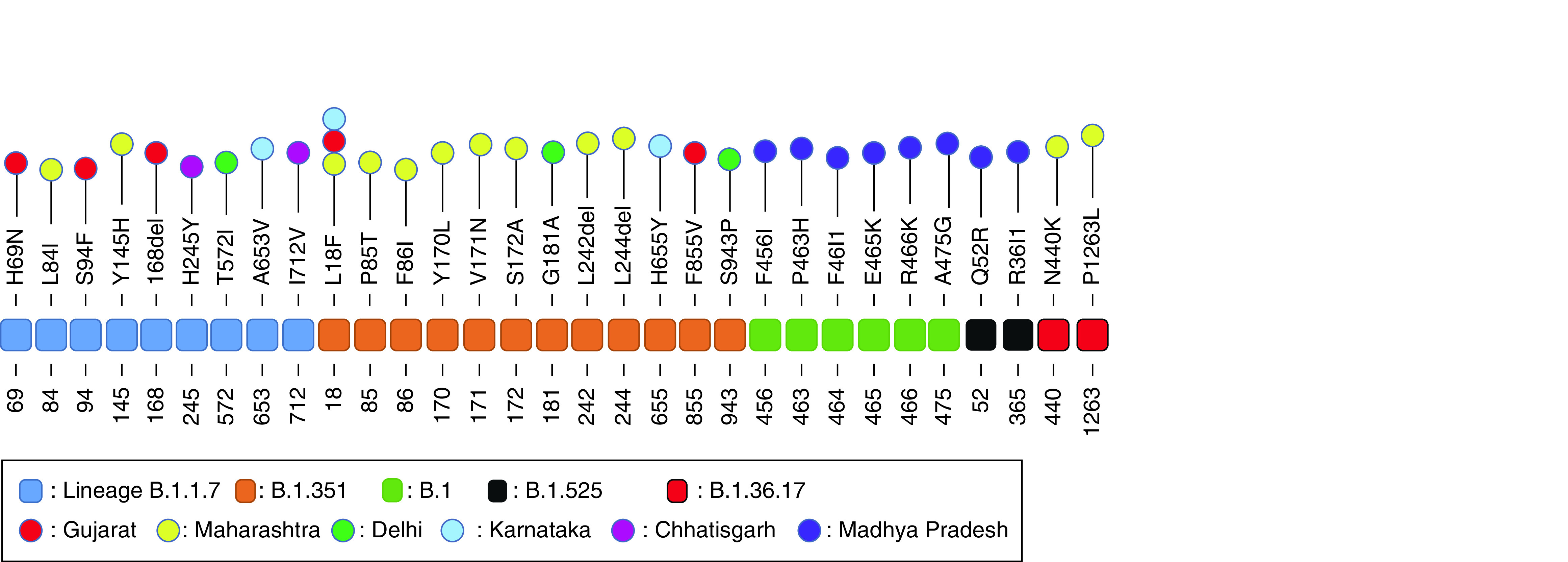
Newly evolved mutations in human SARS-CoV-2 at various spike protein sites in Indian states.

**Figure 8.**
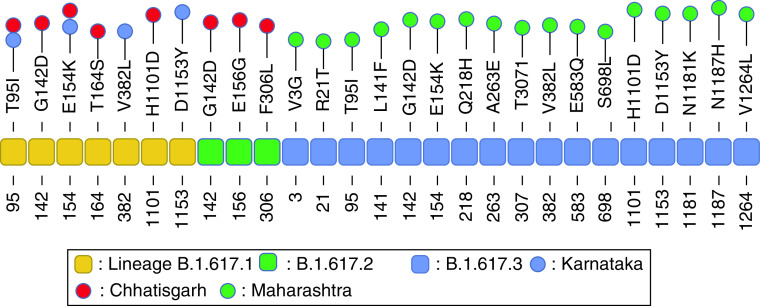
Newly evolved mutations in human SARS-CoV-2 at various spike protein sites in Indian states.

State-wise compilation of observed spike protein mutations types and sites for 10 Indian states is presented in Table 2. The characteristic signature mutations of these lineages are listed in Table 3 [15]. We observed a significant spike protein evolution and all the signature mutations of a particular lineage were not observed in all its spike proteins and rather 51 novel mutations V3G, R21T, L18F, Q52R, H69N, L84I, P85T, F86I, S94F, T95I, L141F, G142D, Y145H, E154K, E156G, T164S, 168del, Y170L, V171N, S172A, G181A, Q218H, L242del, L244del, H245Y, A263E, F306L, T307I, R365I, V382L, N440K, F456I, P463H, F464I, E465K, R466K, A475G, T572I, E583Q, A653V, H655Y, S698L, I712V, F855V, S943P, H1101D, D1153Y, N1181K, N1187H, P1263L and V1264L were recorded in the analyzed samples in various states. Figures 7 & 8 illustrate the documentation of novel mutation sites and types showing lineage-wise and state-wise evolution of human SARS-CoV-2 spike protein in India.

**Table 3.** Newly evolved mutations in the analyzed SARS-CoV-2 lineages circulating in India.

Sl no.	PANGO Lineage	Signature mutations	Newly evolved mutations state wise
1	B.1.1.7	del69/70, del144/145, N501Y, A 570D, D614G, P681H, T716I, S982A, D1118H	M: L84I, Y145H G: H69N, S94F, 168del D: T572I K: A653V Ch: H245Y, I712V
2	B.1.351	D80A, D215G, del241/243, K417N, E484K, N501Y, D614G, A701V	M: L18F, P85T, F86I, Y170L, V171N, S172A, L242del, L244del G: L18F, F855V D: G181A, S943P K: L18F, H655Y
3	B.1	D614G	MP: F456I, P463H, F464I, E465K, R466K, A475G
4	B.1.36.17	D614G	M: N440K, P1263L
5	B.1.525	Q52V, A67V, H69/70del, del144/145, E484K, D614G, Q677H, F888L	G: Q52R, R365I D: Q52R
6	B.1.617.1	L452R, E484Q, D614G, P681R, Q1071H	K: T95I, E154K, V382L, D1153Y Ch: T95I, G142D, E154K, T164S, H1101D
7	B.1.617.2	T19R, del157/158, L452R, T478K, D614G, P681R, D950N	Ch: G142D, E156G, F306L
8	B.1.617.3	T19R, L452R, E484Q, D614G, P681R	M: V3G, R21T, T95I, L141F, G142D, E154K, Q218H, A263E, T307I, V382L, E583Q, S698L, H1101D, D1153Y, N1181K, N1187H, V1264L

Ch: Chhattisgarh; D: Delhi; G: Gujarat; K: Karnataka; MP: Madhya Pradesh; M: Maharashtra.

#### Gujarat (G)

A total of 96 mutations were observed in 314 samples analyzed from Gujarat state (Table 2). Forty eight mutations were unique, present just in one spike protein. D614G was the most predominant mutation found in 302 samples. Other frequent spike mutations with their occurrences in parenthesis included L54F (41), H69del (14), V70del (13), R78M (6), Y144del (15), S477N (6), N501Y (20), A570D (15), E583D (14) D614G (302), Q677H (21), P681H (22), A701T (16), T716I (15), P812L (31), S982A (15), H1083Q (17). Nine samples represented Clade O (Wuhan genotype). The preferential amino acid substitutions at specific positions for Gujarat state included M to I and T to I substitutions at three and five spike proteins sequence positions, respectively. There was a lone case where T was substituted to A as T1116A. Among the 12 mutations in the RBD region of the spike protein in the state, the most frequent mutations with their occurrences in the parenthesis included K417N (4), N440K (4), S477N (6), E484K (5), and N501Y (20; Table 2).

After December 2020, in the second wave of infections, three lineages B.1.1.7, B.1.351 and B.1525 were reported in the analyzed samples from the state. The signature mutations for these lineages are given in Table 3. Ten spike proteins of B.1.1.7 lineage showed 10 signature mutations only, while the remaining five showed convergent evolution. In GISAID accessions EPI_ISL_825054 (eight mutations) and EPI_ISL_825059 (10 mutations), H69del and V70del went missing in the former, while, H69del was replaced by H69N and V70del was replaced by 168del in the latter. In accessions EPI_ISL_1544115, EPI_ISL_1544116, and EPI_ISL_1544117, highest 11 mutations each were recorded. While S94F was added as 11th mutation to the set of 10 signature mutations in the first two, T1116A was added to the third, respectively. H69N, S94F and 168del were the three newly evolved mutations in this lineage in the state, which were absent in the signature mutations that defined the lineage.

Lineage B.1.351 was characterized by eight signature mutations (Table 3). In all the analyzed samples in the Gujarat, seven out of eight mutations excluding del241/243 were observed. It was replaced by L18F in GISAID accession EPI_ISL_1704309 and EPI_ISL_1703836. In the latter spike protein, an additional F855V mutation was also noted taking the number of maximum mutations recorded to nine for this lineage. L18F and F855V were the novel mutations that arose in the state in this lineage.

In the lineage B.1.525 in Gujarat, the characteristic signature mutation del144/145 was not observed in the analyzed samples, rather a new mutation arose as R365I. Q52V of the signature mutation changed to Q52R (Table 3).

#### Maharashtra (M)

A higher number of 106 mutations were reported in 156 analyzed spike proteins from the state of Maharashtra that abated the most severe second wave of infections in the country (Table 2). There were 70 unique mutations that were present just in only one spike protein each, while 15 frequently occurring mutations according to their total number of occurrences included S12F (5), L18F (14), T95I (12), E154K (20), E484Q (21), N501Y (14), A570D (9), D614G (152), Q677H (14), P681H (10), P681R (17), T716I (9), S982A (9), H1101D (8) and D1118H (9). Mutation D614G was conspicuously present in 152 samples, while four samples represented clade O (Wuhan clade) and did not show any mutation. Among the 12 mutations in the RBD region of the spike protein in the state (Table 2), the most frequent mutations with their occurrences in the parenthesis included K417N (5), E484K (7), E484Q (21) and N501Y (14).

After December 2020, in the second wave of infections, more transmissible and virulent lineages B.1.617, B.1.1.7, B.1.351 and B.1.36.17 became visible in the state. In 19 samples of lineage B.1.617, one of the signature mutations D1071H (Table 3), was not been observed in the state. Additionally, many new substitutions were observed at various sites. E154K and T95I were the most frequent combination visible in 15 of the 19 samples until February 2021. After which, yet another new mutation H1101D started becoming visible. Interestingly, T95I was not seen after February and never in combination with H1101D in our samples. The total number of mutations ranged from six to eight in this lineage. Four of the signature mutations L452R, E484Q, D614G and P681R were observed in all the samples. Additional mutations included V3G, R21T, L141F, G142D, E154K, Q218H, T307I, E583Q, S698L, V1060L, D1153Y, N1187H and V1264L (Table 3, Figures 7 & 8).

In the lineage B.1.1.7, nine samples were analyzed. Except del 69/70 that was observed in two analyzed samples remaining seven signature mutations were present in all the samples. The total number of mutations ranged from seven to ten additional mutations included L84I, Y144del and Y145H (Table 3).

Lineage B.1.351 is characterized by eight signature mutations. The number of observed mutations in the analyzed samples varied from five to thirteen. Sample EPI_ISL_1704062, showed the highest mutations (13) among all the analyzed samples, across 10 states. Sample 1703656 showed minimum (5) signature mutations, i.e; K417N, E484K, N501Y, D614G and A701Y, which were present in all the samples. Additional substitutions included L18F, P85T, F86I, Y170L, V171N, S172A, L242del and L244del (Table 3).

The lineage B.1.36.17, contained two new mutations N440K and P1263L along with the signature mutation D614G (Table 3).

#### Karnataka (K)

A total of 44 mutations with 18 unique mutations were observed in 61 analyzed spike proteins (Table 2). Clade O with no mutations with respect to the Wuhan reference sample was visible in seven samples. While the frequently occurring mutations included N440K (5), L452R (5), E484K (6), E484Q (5), D614G (52), N501Y (12), A570D (7), P681H (11), T716I (9), S982A (7), Q1071H (5) and D1118H (7), the most frequently occurring mutation D614G was absent in eight analyzed spike proteins. Among the 7 mutations in the RBD region of the spike protein in the state (Table 2), the most frequent mutations with their occurrences in the parenthesis included N440K (5), L452R (5), E484K (6), E484Q (5) and N501Y (12).

Four lineages B.1.1.7, B.1.525, B.1.351 and B.1.617 surfaced in the state in December 2020. Sub-lineage B.1.617.1 only of Indian variant was visible in our analyzed samples from the state. The highest total number of observed mutations in Karnataka in the first wave of infections and after introduction of above lineages in the second wave was three and ten per spike protein, respectively.

In five out of nine spike proteins belonging to lineage B.1.1.7, all ten signature mutations of the lineage were observed. While in the remaining two spike proteins (accession ID EPI_ISL_159589 and EPI_ISL_1595874) three each (N501Y, P681H and T716I), seven (EPI_ISL_1818595, H69del, V70del and Y144del absent) and eight (EPI_ISL_1818647, H69del, V70del, Y144del absent and A653V additional present) mutations were documented (Figure 7).

Only sub-lineage B.1.617.1 of the Indian variant B.1.617 was visible in our analyzed samples in the state of Karnataka. The total number of mutations ranged from five to seven. Five signature mutations L452R, E484Q, D614G, P681R and Q1071H were observed in all the samples. Additional amino acid substitutions included T95I, E154K, V382L, and D1153Y (Table 3 & Figure 8).

Lineage B.1.525 uniformly showed five spike protein amino acid substitutions namely Q52R, E484K, D614G, Q677H, and F888L, and no additional substitutions were observed in the analyzed samples.

Lineage B.1.351 spike proteins showed either seven or eight amino acid substitutions each. Seven signature mutations namely D80A, D215G, K417N, E484K, N501Y, D614G and A701V were present in all the samples while the additional new substitutions included L18F and H655Y (Table 3 & Figure 7).

#### Delhi (D)

A total of 35 mutations were observed in 60 analyzed spike proteins in Delhi (Table 2). There were 35 unique mutations and clade O was observed in 9 samples (Table 1). D614G was present in all the remaining 51 samples. The other frequent mutations included A570D (12), N501Y (11), P681H (8), S982A (10) D1118H (10). Among the 10 mutations in the RBD region of the spike protein in the state (Table 2), the most frequent mutations with their occurrences in the parenthesis included V367F (2), E484K (4) and N501Y (11).

Three PANGO lineages B.1.525, B.1.1.7 and B.1.351 have been observed in the analyzed Delhi samples. Thirteen samples of lineage B.1.1.7 revealed two (EPI_ISL_1716802) to eight mutations (EPI_ISL_1716813). Seven signature mutations except del69/70 and del144/145 were visible in the state, though not in every sample. Sample EPI_ISL_1716813 contained seven signature mutations and an additional new T572I mutation. In the remaining samples, for instance, four, three and two samples contained seven, six and two of the signature mutations, respectively, while three, four, and five mutations were represented in one sample each only (Tables 2 & 3).

In lineage B.1.525 samples, number of mutations recorded ranged from four to five. Five of the signature mutations analyzed were A67V, D614G, E484K, Q677H and F888L. del69/70 and del144/145 were not observed (Table 3). At 52 spike protein sequence site, V (Valine) of the signature mutation was substituted by R (Arginine). Q52R was hence a new substitution (Table 3 & Figure 7). Total number of reported mutations was nine for the South African lineage B.1.351. Seven signature mutations except del241/243 were visible in the analyzed samples. Additionally new substitutions G181A and S943P were also observed (Table 3 & Figure 7).

#### Chhattisgarh (CH)

Forty-three samples analyzed from the state showed 38 mutations. Most frequent mutations included L452R (7), N501Y (14), A570D (14), D614G (41), P681H (15), T716I (14), S982A (15), and D1118H (14) (Table 2). There were 18 unique mutations and D614G was found to be absent in samples EPI_ISL_1173164, and EPI_ISL_1173162. clade O was not observed in the analyzed samples in Chhattisgarh. Preferential amino acid substitutions from T to I was observed in T95I (3), T678I (2), T716I (14), while T (Threonine) also changed to K (Lysine) and R (Arginine) as T478K (2) and T19R (1), respectively. Likewise, Q (Glutamine) to H (Histidine) substitutions were observed at spike sequence positions Q613H (1), Q1071H (3). Among the six mutations in the RBD region of the spike protein in the state, the most frequent mutations with their occurrences in the parenthesis included L452R (7), E484Q (5), and N501Y (14) (Table 2).

During the second wave of infections, two lineages B.1.617 and B.1.1.7 were detected in the analyzed samples. The Indian variant was represented as two sub-lineages B.1.617.1 and B.1.617.2. A total of five to eight mutations with five signature mutations L452R, E484Q, D614G, P681R, and Q1071H were present in all samples of B.1.617.1 sub-lineage. Additionally new amino acid substitutions T95I, G142D, E154K, T164S and H1101D were observed (Table 3). Sub-lineage B.1.617.2 showed six to ten (EPI_ISL_ 1731755) mutations. All the seven signature mutations were observed in the state with additional new amino acid substitutions listed as G142D, E156G and F306L (Table 3 & Figure 8).

Fourteen samples of lineage B.1.1.1.7 showed seven to eleven mutations. Eleven samples showed seven of the signature mutations of the lineage, while with an additional new substitution I712V, this number rose to eight in two samples. H69del, H70del, Y144del and a new H245Y were the additional mutations recorded in the sample with highest 11 mutations (EPI_ISL_1731751) in the state. H245Y, I712V were the new amino acid substitutions recorded in the state in this lineage (Table 3 & Figure 7).

#### Telangana (T)

Forty-two samples revealed 33 amino acid substitutions in the state of Telangana. The total mutations varied from one to ten. Clade O was present in five samples. The most frequent mutation D614G, present in 35 samples was not detected in samples EPI_ISL_850514 and EPI_ISL_495164. Preferential amino acid substitutions were visible as T to I (T716I) and M to I substitutions (M731I, M1229I). The seven mutations in the RBD region of the spike protein in the state with their occurrences in the parenthesis included P384L (1), K417N (1), N440K (2), E484D (1), E484K (2), N501Y (5) and A520S (3) (Table 2).

#### Madhya Pradesh (MP)

Thirty-one samples showed 21 amino acid substitutions in the spike protein. Nineteen mutations were unique and four samples depicted clade O. D614G was the most frequent mutation observed in 26 samples. Preferential amino acid substitution in terms of change of F to I was observed at two spike sequences F456I and F464I. Lineage B.1 seen in the state encompassed mutations as F456I, P463H, F464I, E465K, R466K, A475G, D614G showing substitution of four successive AAs (463 to 466) in a single sample (EPI_ISL_1251265).The nine mutations in the RBD region of the spike protein in the state with their occurrences in the parenthesis included S399stop (1), F456I (1), P463H (1), F464I (1), E465K (1), R466K(2), S469T (1), A475G (1), N501Y (1) (Table 2).

#### Rajasthan (R)

Twelve mutations were detected in 16 samples and five mutations were unique. Clade O was found in four samples. D614G was the most common mutation found in 12 samples. N440K was the only mutation located in RBD of the spike protein (Table 2).

#### Haryana (H)

Three mutations observed in 18 samples with clade O represented in three samples, D614G was the dominant mutation detected in 15 samples. P330S was the only mutation located in RBD of the spike protein (Table 2).

#### Uttar Pradesh (UP)

The analyzed 20 samples from the state revealed seven mutations. There were six unique mutations and clade O was detected in five samples and D614G was the most frequent mutation that was present in all the remaining samples. A522S was the only mutation located in RBD of the spike protein (Table 2).

## Discussion

As the SARS-CoV-2-induced pandemic expanded, research efforts around the world started focusing on whole-genome sequencing, understanding epidemiology, documenting genetic diversity, analyzing the evolving mutations and their effects, monitoring the genomic evolution of the virus and developing diagnostics, therapeutics and vaccines. Detailed mutation analysis of human SARS-CoV-2 spike proteins sampled between March 2020 to August 2021, involving both the first and second waves of COVID-19 infections from ten different Indian states in the present analysis revealed both a rise in number as well as the induction of many novel functionally significant mutations in the SARS-CoV-2 spike protein in India. Introduction of global lineages, the emergence of local variant strains and induction of new mutations in the existing strains with the passage of time and laxity of COIVD-19 appropriate behavior contributed to this upsurge. The number of spike mutations in various states varied from one to five during the first wave of COVID-19 infections, while a sudden spurt in the frequency of spike protein mutation took the maximum number to as high as thirteen per spike protein during the second wave of infections.

Since its emergence in 2019, the SARS-CoV-2 virus has been continuously evolving and has resulted in many novel lineages that emerged in different parts of the world. During the first wave of infections in India until September 2020, lineage B.1 with D614G mutation was visible in the analyzed samples. Later, between October and December 2020, lineage B.1.1.7 also became visible with signature mutations del69/70, del144/145, N501Y, A 570D, D614G, P681H, T716I, S982A and D1118H. While, from January 2021 onwards, eight SARS-CoV-2 lineages B.1.1.7, B.1, B.1.351, B.1.525, B.1.36.17, B.1.617.1, B.1.617.2 and B.1.617.3 could be observed in our representative sample. Lineage B.1.617 popularly known as the ‘Indian variant’ with its three main sub-lineages B.1.617.1 (Kappa), B.1.617.2 (Delta) and B.1.617.3 first emerged in India in December 2020. B.1.617.2 sub-lineage with the basic reproductive number (Ro) of 5.08 versus 2.79 for Wuhan-Hu-1 strain outcompeted pre-existing lineages B.1, B.1.1.7 (Alpha) and B.1.617.1 (Kappa). With predominant mutations T19R, del157-158, K417N, L452R, T478K, E484K/Q, P681R and D950N [15], the Delta variant showed higher transmissibility and risk of hospitalization [16–18] and fuelled the massive devastation associated with the second wave of COVID-19 infections in India.

The further evolution in the prevalent global and local lineages in the analyzed samples was seen to be propelled by the induction of additional novel mutations which were visible both in terms of increased number and their diverse types. The maximum number of 13 mutations per spike protein was documented in the state of Maharashtra which battled a brutal and long phase of COVID-19 infections. From January to August 2021 (second wave) a total of 51 new mutations besides the listed signature mutations evolved in eight prevalent lineages in 763 samples from ten Indian states included in the present study. The emergence of many new mutations in addition to the signature mutations of specific lineages indicates a convergent evolution in the virus that might be responsible for an increase in transmission and pathogenesis. The selection pressure resulting from the rollout of the vaccination program in January 2021 along with other factors might have triggered the emergence of novel spike mutations in India.

Several studies have documented the role of naturally induced mutations in RBD and other regions of spike protein in viral entry, transmission, infectivity, pathogenesis and immune escape [7,19,20,21]. VOCs Alpha and Delta showed higher transmission rate and spread globally and VOCs Alpha and Beta were discovered to be resistant to neutralizing antibodies, thereby, affecting the effectiveness of vaccines [22].

In the presently analyzed dataset, maximum number of mutations (43%) were recorded in SI^A^ domain followed by SI^B^ (14.5%) and β-sheet (6.9%). The SI^A^ spike domain includes the N-terminal domain (NTD), RBD and the receptor binding motif (RBM). These three important regions of spike protein have been the primary targets of vaccines and antibody-based therapeutics including monoclonal antibodies (mAbs), polyclonal antibodies and convalescent plasma. The spike RBD has undergone extensive evolution with the passage of time and as many as 824 RBD mutations have already been documented [23]. In this study, the RBD region including the RMF revealed 35 distinct mutation types located at 31 distinct sites as A344T, R346T, R365I, V367F, S373L, D377Y, V382L, P384L, S399stop, K417N, T430I, N439K, N440K, L452R, F456I, P463H, F464I, E465K, R466K, S469T, A475G, S477I, S477N, T478K, E484D, E484K, E484Q, Q493stop, S494P, N501Y, Y508H, L517H, A520S, A522S and A522V. Many of these mutations have known functions to contribute to increased pathogenesis, transmission and immune evasion.

In the context of spike RBD mutations, Delta sub-lineages characteristically contain two mutations each in the spike RBD and are commonly known as double mutants. B.1.617.1 (Kappa) and B.1.617.3 contain L452R and E484Q mutations each, while B.1.617.2 (Delta) has L452R and T478K as the RBD mutations [24]. The L452R mutation is associated with increased infectivity by enhanced interaction between RBD and hACE2 receptor [25]. Co-operatively, T478K and T452R mutations stabilize the RBD–ACE2 complex to increase the rate of virus infectivity and affect the immune response [20]. Another important spike mutation P681R leads to increased furin cleavage leading to greater infectivity and higher viral loads. Together, this cocktail of B.1.617.2 mutations imparts higher transmissibility, infectivity and immune evasion potential [20,25–27].

In the present samples, D614G has been the most widely occurring mutation, present in 697 out of the 763 analyzed viruses. It has been shown to result in significantly higher transmission and host infectivity [28]. The other frequent mutations observed were N501Y, P681H, A570D and D1118H observed in 77, 70, 60 and 59 of the analyzed 763 viruses, respectively. With respect to the state wise distribution of mutations, five most frequent spike mutations sites with occurrences in various states included 501 (G, M, K, D, CH, T and MP), 484 (G, M, K, D, CH and T), 440 (G, M, K, D, CH and T), 452 (M, K, and CH) and 417 (G, M, K, D and T), respectively. Interestingly, we observed state-specific unique set of mutations in six out of ten analyzed states that showed up as A344T, R346T, R365I, N439K, Q493stop, S494P and Y508H in Gujarat; S373L and L517H in Maharashtra; V367F, D377Y and A522V in Delhi; P384L and E484D in Telangana; S399stop, F456I, P463H, F464I, E465K, R466K, S469T and A475G in Madhya Pradesh and A522S in Uttar Pradesh. The differential level of infections, pathogenicity and transmission in different states could possibly be due to these state-specific mutations. Functional characterization of many of the presently observed mutations has been illustrated in many studies [20,21,27–32]. Most of the RBD mutations strengthen the RBD–ACE2 binding supporting the evolution of the virus to more infectious variants [21,27,29]. Mutations N501Y, D614G and others have been found to be associated with reinfection, partial resistance to vaccines and increased transmissibility. Mutation V367F, N354D and T478K in SARS-CoV-2 in RBD has been associated with enhanced hACE2 binding affinity and increased viral infectivity [21,27,29]. Mutations L452R and E484Q found in Indian variants have been shown to disrupt the binding between RBD and many known antibodies leading to vaccine escape [21]. Cherian *et al.* 2021 [20] attributed the higher pathogenicity, transmission and acute infections of SARS-CoV-2 in the state of Maharashtra to the presence of spike mutations L452R, T478K, E484Q and P681R. Further, Wang *et al.* [21] identified R403K, K417N/T, L452R, A475S, E484K, F486L, F490S/L, Q493L and S494P as most likely vaccine escape mutations. The S1/S2 cleavage site mutations H655Y, N679K and P681H result in increased S1/S2 furin cleavage and facilitate efficient entry into the host [28,30–32]. Many spike mutations have been shown to affect the neutralization ability of monoclonal and polyclonal therapeutic and convalescent antibodies. While mutation N439K resulting in increased binding affinity with ACE2 receptor was observed to neutralize the monoclonal and polyclonal antibodies in people who recovered from infection [31,33], deletion mutation Y144del was observed to modulate the effects of neutralizing antibodies [30,34]. Likewise, mutations K417N/T, N439K, L452R, Y453F and N501Y have been listed to be the most significant immune escape spike RBD mutations [35]. Further, multiple spike substitutions at 477 (S477G, S477N and S477R) and 484 positions (E484A, E484D and E484K) have characteristically shown resistance toward convalescent sera [28,31,35].

Interestingly, amidst the presence of so many mutant and more transmissible and virulent lineages, clade O (Wuhan isolate) was visible in India throughout the assessment period from March 2020 even until 2021. As many as 50 out of 763 analyzed spike proteins were observed representing clade O with no mutations observed in them relative to the human SARS-CoV-2 spike protein reference sequence from Wuhan, China. These samples were present in all the analyzed states except Chhattisgarh. A maximum of nine samples each were present in Gujarat and Delhi, while Haryana had the minimum number of three. Also, the initial lineage D614G co-existed in the population and remained visible throughout.

One of the most significant revelations of the present analysis has been the identification of spike protein regions S1^B^–S1^C^ and S1^C^–S1^D^ linkers and fusion peptide domains with no mutations. This information is important amidst reports of a significant reduction in vaccine efficacy and antibody neutralization against the spike protein-based vaccines or monoclonal therapeutic antibodies [30,31,36]. In this context, the above-mentioned conserved spike regions can be important potential targets for developing future diagnostics, therapeutics or vaccines.

## Conclusion

The analysis provides useful insights into the distribution of accrued mutations across the spike protein, their density in different regions of spike protein, the frequency and state-wise occurrence(s) of particular mutations, identification of mutation-rich, mutation-poor and mutation-nil regions in the spike protein and most importantly, comparison of spike protein mutations during first and second waves of infections in India to understand the evolutionary trajectory of SARS-CoV-2 in India. The spike protein now has an expanded mutational landscape and its evolution seemingly has facilitated the transmission of the virus by modulating ACE2 receptor binding affinity or immune evasion leading to reduced efficacy of vaccines and therapeutic antibodies. The continuous monitoring of emerging variants through mutation analysis and functional characterization of the induced mutations is important to track the further evolutionary course of the virus and to understand the effects of mutations on viral epidemiology. This information will eventually help in devising intervention strategies. In the context of the characterized spike RBD mutations favoring increased transmission, pathogenesis, immune evasion and reduced neutralization, it is probably time to possibly look at the more conserved parts of the spike protein or other parts of the virus for designing the next-generation therapeutics and vaccines.

**Summary points**

- The present study tracks the evolutionary trajectory of SARS-CoV-2 spike protein during the first and second waves of COVID-19 in India.
- A detailed mutation analysis of spike protein of 763 virus samples taken from Global Initiative on Sharing All Influenza Data belonging to 10 Indian states between March 2020 to August 2021 revealed the presence of 242 mutations corresponding to 207 spike sites. The mutations were observed to be differentially distributed across states and domains of spike protein.
- The highest number of mutations occurred in S1^A^ domain. Interestingly, no mutations were detected in S1^B^–S1^C^ and S1^C^–S1^D^ linkers and fusion peptide domains.
- The number of detected mutations rose from five per spike protein during the first COVID-19 wave to thirteen during the devastating second wave of COVID-19 infections in India.
- The five most frequent mutations included D614G, N501Y, P681H, A570D and D1118H.
- 51 novel mutations emerged in the circulating lineages during the assessment period in 10 Indian states. Many of the incurred mutations have previously been functionally characterized to show immune evasion and resistance to antibody neutralization.
- The evolution of spike protein in analyzed Indian states seems to be fuelled by introduction of global lineages, emergence of local lineages (Delta and its sub-lineages) and induction of novel co-occurring mutations.
- The S1^A^ domain that includes the N-terminal domain, receptor binding domain and receptor binding motif is heavily mutated. This is a matter of concern as these three important regions of spike protein have been the primary targets of vaccines and antibody-based therapeutics including monoclonal antibodies (mAbs), polyclonal antibodies and convalescent plasma.
- Many of the incurred mutations have been previously characterized to show immune escape and resistance to neutralization by antibodies. In this context, importantly, the study has identified mutation rich and no mutation regions in spike protein. These conserved spike regions can be useful for designing future diagnostics, vaccines and therapeutics.
